# Three cases of a peripheral primitive neuroectodermal tumor diagnosed using computed tomography or magnetic resonance imaging

**DOI:** 10.3892/ol.2013.1463

**Published:** 2013-07-15

**Authors:** JUNXIA LI, PEIYOU GONG, ZHUSHI GUANG

**Affiliations:** 1 Department of Oncology, Yantai Yuhuangding Hospital, Yantai, Shandong 264000, P.R. China; 2 Department of Radiology, Yantai Yuhuangding Hospital, Yantai, Shandong 264000, P.R. China

**Keywords:** peripheral primitive neuroectodermal tumor, computed tomography, magnetic resonance imaging

## Abstract

The present study describes the findings from three cases of peripheral primitive neuroectodermal tumors (PNETs) diagnosed using computed tomography (CT) or magnetic resonance imaging (MRI). The patients were all diagnosed with PNETs of the peripheral central nervous system. All the lesions were soft-tissue masses with cystic degeneration. The CT images revealed that the lesions were large and inhomogeneous, with unclear borders and cystic degeneration. The surrounding tissues and structures were compressed against each other. Following the enhancement of the CT, the solid components of the tumors were enhanced, whereas the cystic components did not show enhancement. Following the enhanced MRI, irregular enhancement was noted in the solid components and the cystic and solid masses showed unclear borders. The surrounding tissues were compressed. The solid components of the tumors were enhanced, whereas the cystic components showed no enhancement. Based on these observations, PNETs were diagnosed. Thus, a CT or MRI is mandatory for the precise diagnosis of a peripheral PNET.

## Introduction

A peripheral primitive neuroectodermal tumor (PNET) is a rare disease that was first recognized by Arthur Purdy Stout in 1918 and is classified within the family of small round cell tumors (1). PNETs are more prevalent in children than in adults and occur more often in the central nervous system than in the peripheral areas. A peripheral PNET is similar to an Ewing’s sarcoma (2). Several studies have described the imaging findings of peripheral PNETs (3,4). However, the three cases that are reported in the present study are all adults with lesions that occurred in the abdominal cavity and at the base of the anterior cranial fossa, which is rare. The purpose of the present study is to characterize the assessments of these rare peripheral PNETs using computed tomography (CT) or magnetic resonance imaging (MRI). Written informed consent was obtained from the patients.

## Case reports

### Case 1

**Figure 1:**
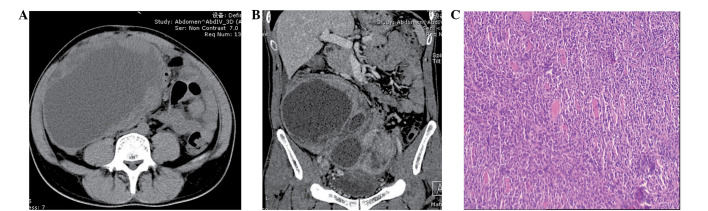
Case 1. (A) A large cystic and solid mass was evident in the abdominal cavity and large liquefaction necrosis areas were observed inside. (B) The solid components of the tumor were significantly enhanced. The surrounding vessels were compressed and shifted from the usual position. (C) According to the pathological imaging, the tumor showed highly cellular sheets of small, round cells with hyperchromatic nuclei (HE staining; magnification, ×100).

A 51-year-old male with a mass in the abdominal cavity was referred to the Yantai Yuhuangding Hospital (Yantai, Shandong, China). Following a CT examination, a plain scan revealed cystic and solid masses in the abdominal cavity with large low-density areas. The largest mass measured ~19×9×13 cm. The masses involved the mesentery, omental bursa and retroperitoneum, with uneven density and a CT value of 19–46 HU (Fig. 1A). Subsequent to scanning enhancement, the solid components of the masses exhibited mild to moderate inhomogeneous enhancement with membrane separation (Fig. 1B) and encased the mesenteric vessels and portal veins. No clear border was observed between the local masses and the surrounding organs. The patient was hospitalized for an abdominal tumor resection. During the surgery, the border between the masses and the surrounding tissues and structures remained unclear and the surface of the masses was speckled. The post-operative pathology report indicated a PNET (Fig. 1C). The patient received systemic chemotherapy following surgery; however, 4 months later, multiple pulmonary metastases were detected. Despite receiving salvage chemotherapy, the patient succumbed 10 months after the diagnosis.

### Case 2

**Figure 2:**
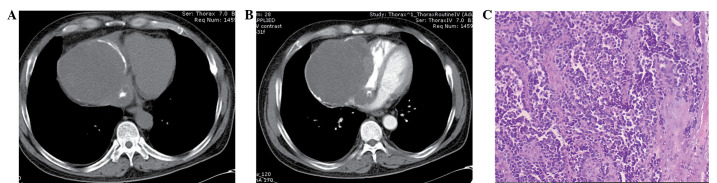
Case 2. (A) A cystic hypodense shadow was observed on the right side of the pericardium, with calcification of the cyst wall. (B) Subsequent to scanning enhancement, the lesions did not show significant enhancement. The pericardium, the source of the lesions, invaded the right ventricular wall and protruded into the chambers of the heart. (C) The tumor exhibited a malignant neoplasm composed predominantly of small cells (HE staining; magnification, ×100).

A 53-year-old female with precordialgia and chest tightness was referred to the Yantai Yuhuangding Hospital. No obvious abnormality was identified on the clinical laboratory tests. Using a CT examination that was performed following hospitalization, a plain scan was performed on the right side of the pericardium to obtain an image of the cystic hypodense shadow, in which a homogenous density, a thicker cyst wall and calcification were observed (Fig. 2A). Subsequent to scanning enhancement, the lesion wall changed significantly, but the middle portion of the lesion did not show significant enhancement. The lesion invaded the right ventricular wall and protruded into the right ventricle cavity (Fig. 2B and C). After assessing the results of the pre-operative examination, a thoracotomy was performed on the patient in order to resect the right pericardial mass. The mass resembled fish flesh and was ~17×16×18 cm in size. The cut surface of the mass was broken and jagged and displayed necrosis. Post-operative immunohistochemistry revealed small, round, blue-stained cells that were tightly arranged. Based on the combined results of the morphology and immunohistochemistry, the diagnosis of a primitive PNET was considered (Fig. 2C). The patient had no further treatment and had no complications. The patient was well in the 6-month follow-up after surgery.

### Case 3

**Figure 3:**
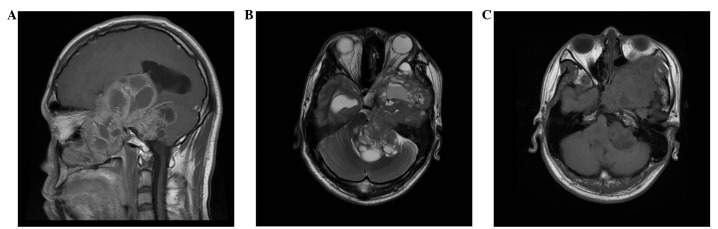
Case 3. (A) The T1-weighted soft-tissue mass was wide and large at the base of the anterior cranial fossa, and the surrounding tissues were extensively destroyed. (B) T2-weighted signal imaging occurred in the mass. (C) The solid components of the tumor were significantly enhanced.

A 26-year-old male with exophthalmos was referred to the Yantai Yuhuangding Hospital. The MRI examination revealed a mass at the base of the anterior cranial fossa involving the right orbital, sphenoid and pituitary glands. The mass invaded the intracalvarium, in which the four ventricles were deformed by compression. The mass displayed a T1-weighted image (T1WI) signal, compared with the muscle, which represented an area of bone destruction or bone marrow replacement (Fig. 3A). At a higher T2WI signal, a cyst was observed (Fig. 3B). Following the enhanced MRI, the solid components of the tumor demonstrated inhomogeneous enhancement (Fig. 3C). Following hospitalization, the patient underwent a resection for a biopsy of the mass at the local skull base. The sections were stained with routine hematoxylin and eosin (HE) and the report indicated a PNET. The patient received regional radiotherapy following surgery. Three months later, the patient developed hydrocephalus and the cerebrospinal fluid was positive for neoplastic cells. No other treatment was administered and the patient succumbed.

## Discussion

A PNET has a high degree of malignancy and recurrence and a poor prognosis. In clinical practice, this tumor is a rare malignant disease that rapidly enlarges in a short period of time, compressing the surrounding structures and invading the adjacent tissues to cause local symptoms. Light microscopy reveals that the histopathological features of a PNET are cells that are arranged like a nest and tumor cells that are round or oval with a deep-staining nucleus. Less cytoplasm and an unclear border are also observed. Homer-Wright rosettes are usually visualized. Necrosis and mitotic activity are easy to identify (5). Weissferdt and Moran (6) reported that PNETs express cytokeratin and S100 and show a positive rearrangement for the EWSR1 gene at the 22q12 locus. Mandal *et al*(7) reported that PNETs immunohistochemically express CD99, synaptophysin (syn) and neuron-specific enolase (NSE).

Another previous study reported that peripheral imaging shows PNET to have a lack of characteristics (8). The majority of these studied clinical cases have involved children and adolescents (3,7,8). The characteristics of a PNET, as shown on CT and MRI, are as follows: i) A large soft-tissue mass, often >5 cm in size, that is lobulated, with the accumulated fusion of multiple soft tissues. The surrounding organs are compressed and the border is unclear, displaying malignant features. ii) The density or signal is uneven due to the rapid growth of the mass, and ischemic necrosis and cystic degeneration may occur when the blood supply is lacking. A centric necrotic mass or cystic solid mass is often observed with evident easy bleeding, which may be related to a rich blood supply. Calcification is rare and, if present, is mottled and may be associated with intratumoral hemorrhage organization. iii) The degree of enhancement ranges from mild to significant and the enhancement of the separated samples are unique in PNETs compared with other malignant tumors. The majority of the blood vessels are wrapped.

The three cases in the present study shared certain similar imaging observations. The cystic and solid masses were large and certain masses were accompanied by calcification. No clear border was evident between the masses and the surrounding tissues. The surrounding organs were compressed and shifted from the usual position. Subsequent to enhancement of the CT or MRI, the solid components of the mass showed significant inhomogeneous enhancement. These observations were similar to those of previous studies (4). However, the predilection site of the PNET in the present cases was unique, particularly for case 2, in which the pre-operative diagnosis was of a pericardial mesothelioma. The common characteristics shared between a pericardial mesothelioma and a PNET include a liquefaction necrosis area in the tumor tissue and calcification of the tumor wall. No similar case report was identified in the literature.

In conclusion, CT and MRI examinations clearly show the predilection site of a PNET, the internal structure of the tumor, anatomical associations between the tumor and adjacent nerve and vascular structures, tumor recurrence and distant metastasis. In the present case reports, the predilection sites of the cases were unique and their imaging observations shared certain similarities that may provide reference for similar clinical diagnoses in the future.
